# From Cure to Complexity: Post‐SVR Liver and Metabolic Trajectories in Diabetic Patients

**DOI:** 10.1111/liv.70631

**Published:** 2026-04-17

**Authors:** Clelia Asero, Maria Stella Franzè, Teresa Maltese, Alberto La Spada, Daniele Lombardo, Claudia Grisanti, Giuseppina Russo, Annalisa Giandalia, Concetta Pitrone, Roberto Filomia, Gaia Caccamo, Carlo Saitta, Anna Licata, Teresa Pollicino, Irene Cacciola

**Affiliations:** ^1^ Department of Clinical and Experimental Medicine University of Messina Messina Italy; ^2^ Medicine and Hepatology Unit University Hospital of Messina Messina Italy; ^3^ Unit of Nephrology and Dialysis University Hospital of Messina Messina Italy; ^4^ Division of Advanced Diagnostic Laboratories University Hospital of Messina Messina Italy; ^5^ Department of Human Pathology of Adulthood and Childhood “G. Barresi” University of Messina Messina Italy; ^6^ Internal Medicine and Hepatology, PROMISE University Hospital of Palermo Palermo Italy

**Keywords:** diabetes mellitus, HCV, hepatocellular carcinoma, liver cirrhosis, MBOAT7, single nucleotide polymorphisms

## Abstract

**Background and Aims:**

The long‐term impact of HCV cure on hepatic and metabolic outcomes in patients with type 2 diabetes (T2D) remains insufficiently defined. This study evaluated T2D‐related vascular complications, liver disease progression and overall survival over 9 years of follow‐up, also exploring genetic variability contribution.

**Methods:**

Consecutive T2D patients with HCV‐related chronic liver disease or cirrhosis treated with direct‐acting antivirals (DAAs) between 2015 and 2018 at the University Hospital of Messina were prospectively followed until September 2024. Demographic, biochemical, and clinical data were collected at baseline and throughout follow‐up. Regression models were applied to identify predictors of metabolic and hepatic outcomes. Genetic variants—*PNPLA3* rs738409, *TM6SF2* rs58542926 and rs641738 at the *MBOAT7/TMC4* locus—were also assessed.

**Results:**

A total of 183 patients (52% males, median age 67 years; 56% cirrhotic) were followed for a median of 48 months (range 24–84). Despite significant improvements in HbA1c (*p* = 0.006), liver‐stiffness (*p* < 0.001), gamma‐globulins (*p* < 0.001), and aminotransferases (*p* < 0.001), only 27.3% maintained clinical stability. Liver disease progression occurred in 20.8% of patients and was related to cirrhosis (*p* = 0.021), prior decompensation (*p* = 0.07), and the MBOAT7 variant (*p* = 0.025). Macrovascular and microvascular complications developed in 50.8% and 33.9% of patients, respectively, mostly within 2 years after SVR. In multivariate models, higher TyG index (*p* = 0.038) predicted the composite progression endpoint, while elevated LDL cholesterol (*p* = 0.048), mortality.

**Conclusions:**

Although DAAs lead to metabolic and hepatic improvements, long‐term prognosis in T2D patients remains largely determined by baseline liver disease severity, insulin resistance, and genetic background. These findings emphasize the importance of early antiviral treatment and optimized metabolic management in this high‐risk population.

## Lay Summary

- Direct‐acting antivirals (DAAs) treatment cures HCV infection and improves liver disease natural history, reducing liver decompensation rates and contributing to glycated haemoglobin amelioration in long‐term follow‐up.
- Despite viral eradication, T2D patients with high insulin resistance levels face vascular complication onset and liver disease progression soon after therapy.
- Early treatment of HCV infection is mandatory, as liver cirrhosis and clinically significant portal hypertension drive the onset of liver decompensation and hepatocellular carcinoma.
- The common genetic variant near *MBOAT7* (rs641738) may increase the risk of liver complications, but larger studies are needed to confirm this hypothesis.

**Abbreviations**
ALT: alanine aminotransferase
AMI: acute myocardial infarction
AST: aspartate aminotransferase
BMI: body mass index
CKD‐EPI equation: Chronic Kidney Disease Epidemiology Collaboration equation
CLD: chronic liver disease
CP: Child‐Pugh score
CT: computed tomography scan
DAAs: Direct‐Acting Antivirals
EASL: European Association for the Study of the Liver
eGFR: estimated Glomerular Filtration Rate
FPG: fasting plasma glucose
GGT: gamma glutamyl transpeptidase
GLP1ra: glucagon‐like peptide‐1 receptor agonists
HbA1c: glycated haemoglobin
HBV: hepatitis B virus
HCC: hepatocellular carcinoma
HCV: hepatitis C virus
HDL‐c: high‐density lipoprotein cholesterol
HIV: human immunodeficiency virus
IR: insulin resistance
LD: liver decompensation
LDL‐c: low density lipoprotein cholesterol
LSM: liver stiffness measurement
MACE: major cardiovascular events
MBOAT7 rs641738: membrane‐bound O‐acyltransferase domain‐containing 7
OS: overall survival
PAD: peripheral arterial disease
PCR: real‐time polymerase chain reaction
PNPLA3 rs738409: patatin‐like phospholipase domain‐containing 3
SGLT2i: sodium‐glucose cotransporter 2 inhibitors
SNPs: single nucleotide polymorphisms
SVR: sustained virological response
TD2: type 2 diabetes
TM6SF2 rs58542926: transmembrane 6 superfamily member 2
TyG index: Triglyceride‐Glucose index

## 1 Introduction

Hepatitis C virus (HCV) infection represents nowadays a public health burden as it continues to be one of the most common causes of chronic liver disease and a leading indication for liver transplantation, affecting approximately 71 million people worldwide [1, 2]. It is well established that around 74% of HCV‐infected individuals experience at least one extrahepatic manifestation, which is why HCV infection is now considered a systemic disease [3]. Among endocrine disorders, type 2 diabetes (T2D) is the most frequently associated metabolic condition in HCV‐infected patients [4, 5, 6]. Studies estimate that individuals with HCV present a 3.8‐fold higher risk of developing insulin resistance (IR) and a fourfold increased risk of T2D onset compared to the general population. Moreover, metabolic syndrome occurs in approximately 20%–30% of HCV‐infected patients, supporting the hypothesis of a direct viral role in glucometabolic impairment [7]. However, the impact of HCV infection on low‐density lipoprotein cholesterol (LDL‐c) levels and cardiovascular risk remains controversial [4, 8].

The introduction of Direct‐Acting Antivirals (DAAs) has revolutionized the natural history of HCV‐related liver disease. More than 90% of patients treated with DAAs achieve a sustained virological response (SVR) [9], along with improvements in liver function, serum biomarkers, Child‐Pugh (CP) score and liver stiffness measurement, regardless of the presence of liver cirrhosis [10, 11]. Achieving SVR has been shown to positively impact liver function, as DAAs regimens can halt or even reverse liver fibrosis [12], thereby reducing the risk of liver decompensation and simultaneously improving the overall survival [13]. However, long‐term data on the benefits of SVR for liver fibrosis and hepatic‐related outcomes, including the incidence of hepatocellular carcinoma (HCC) [14], remain limited, largely due to the short follow‐up periods reported in most studies [15, 16]. Considering extra‐hepatic manifestations, HCV eradication via DAAs therapy has been associated with a 50%–60% reduction of incidence of IR, along with the improvement in fasting serum glucose and HbA1c (glycated haemoglobin) levels observed 24 weeks after treatment [17, 18, 19]. However, as with liver‐related outcomes, the available literature does not provide sufficient evidence on the long‐term effects of DAAs therapy on glycaemic control or the development of T2D vascular complications after SVR, since the median follow‐up in most studies ranges from 24 to 96 weeks [20].

Current international guidelines recommend regular follow‐up after SVR in patients with advanced liver disease (Metavir stage F3 or cirrhosis) or in those with risk factors for disease progression, such as T2D, due to the risk of HCC and liver decompensation development [1]. In this context, single‐nucleotide polymorphisms (SNPs) may contribute to the progression of liver disease. Previous studies have examined the influence of *PNPLA3* p.I148M (rs738409), *TM6SF2* p.E167K (rs58542926) and rs641738 at the *MBOAT7/TMC4* locus (often annotated TMC4 p.G17E and linked to reduced *MBOAT7* expression) on liver disease severity and outcomes. However, findings across cohorts remain inconsistent. In particular, the *MBOAT7‐TMC4* rs641738 variant has been linked to hepatic fat accumulation, inflammation and fibrosis progression in patients with chronic liver disease and may act as a modifier of disease severity [21, 22].

The aim of this study is to evaluate the long‐term impact of HCV eradication with DAAs therapy on glycaemic control, liver disease progression, and overall survival (OS) in a cohort of diabetic patients followed for up to 108 months, while also assessing the potential role of SNPs on these outcomes.

## 2 Patients and Methods

### 2.1 Study Cohort Selection

All T2D patients attending the Hepatology and Diabetology Units of the University Hospital of Messina, treated and cured for HCV infection with DAAs between 1 April 2015, and 31December 2018, were prospectively evaluated until 30 September 2024. Inclusion criteria were the presence of chronic liver disease (CLD—grade F3 according to the Metavir classification) or compensated liver cirrhosis (Child Pugh score class A). Exclusion criteria included hepatitis B virus (HBV) and/or human immunodeficiency virus (HIV) coinfection, as well as the presence of HCC and/or liver decompensation (LD)—defined as ascites, hepatic encephalopathy, or gastrointestinal bleeding—at enrolment [23]. The stage of liver disease prior to DAAs treatment was classified as either liver cirrhosis or CLD based on histological findings (where available), ultrasonographic, and biochemical criteria, as well as liver stiffness measurement (LSM) and upper gastrointestinal endoscopy results [24]. Specifically, patients with CLD exhibited LMS values between 10 and 12 kPa, whereas cirrhosis was diagnosed for values exceeding 12 kPa [20]. The diagnosis was further supported by clinical and instrumental criteria, including a platelet count < 150 × 10^3^, distinctive ultrasonographic features (coarse echo pattern, splenomegaly, portal vein diameter > 12 mm, and caudate lobe hypertrophy), endoscopic evidence of oesophageal and/or gastric varices and a history of liver decompensation [25].

Patients who provided informed consent for genetic testing underwent blood sample collection for SNPs evaluation.

### 2.2 Data Collection

Data were collected at two points: baseline (before initiation of DAAs therapy) and the last follow‐up after achieving SVR. All demographic, clinical, and laboratory data were recorded in a digitalized database. Patients' characteristics included age, gender, body mass index (BMI), presence of liver cirrhosis, T2D and lipid‐lowering therapy intake, history of previous HCV treatment with interferon. Biochemical data included serum values of glycated haemoglobin (HbA1c), fasting plasma glucose (FPG), total cholesterol, high‐density lipoprotein cholesterol (HDL‐c) and low‐density lipoprotein cholesterol (LDL‐c), triglycerides, alanine aminotransferase (ALT), aspartate aminotransferase (AST), gamma glutamyl transpeptidase (GGT), albumin, gamma‐globulins, total bilirubin, creatinine. Renal function was assessed as the estimated glomerular filtration rate (eGFR), calculated via the 2021 CKD‐EPI (Chronic Kidney Disease Epidemiology Collaboration) equation, while LDL‐c was calculated using the Friedewald formula [LDL‐c = total cholesterol—HDL‐c–(triglycerides/5)] and TyG index (Triglyceride‐Glucose index) by the following equation: log (fasting triglycerides (mg/dL) * fasting glucose (mg/dL)/2). Liver stiffness measurements (LSM) were performed using the FibroScan instrument (EchoSens, Paris, France) with either M or XL probes after overnight fasting. Additionally, portal vein diameter, spleen diameter and the presence of oesophageal varices were assessed.

### 2.3 Genetic Polymorphisms Analysis

Genomic DNA was extracted from whole blood using the QIAamp DNA Mini Kit (Qiagen, Hilden, Germany) and stored at −80 C. Three single‐nucleotide polymorphisms (SNPs) were examined: *PNPLA3* rs738409 C > G (p.I148M), *TM6SF2* rs58542926 C > T (p.E167K) and rs641738 C > T at the *MBOAT7/TMC4* locus (commonly annotated TMC4 p.G17E). Genotyping was conducted via real‐time polymerase chain reaction (PCR) employing allele‐specific TaqMan probes (Applied Biosystems, Foster City, USA) on a CFX96 optical reaction module (Bio‐Rad, Hercules, USA). The thermal cycling conditions consisted of an initial denaturation at 95°C for 10 min, followed by 40 cycles of denaturation at 95°C for 15 s and combined annealing/extension at 60°C for 1 min. The fluorescence signals (VIC and FAM) specific to each SNPs were detected and reported by the ABI Step One Plus real‐time PCR system (Applied Biosystems). Control samples of known genotypes were also included for each experiment in every 96‐well plate (no template control, homozygous wild‐type, homozygous mutant, and heterozygous).

### 2.4 Assessment of Clinical Events

Clinical events were defined as the onset of liver decompensation, HCC, vascular T2D complications, and death, assessed with the year of occurrence after SVR. Liver decompensation was defined as the onset of ascites and/or hepatic encephalopathy and/or gastrointestinal bleeding. HCC was diagnosed by standard histological and/or radiological findings, according to the EASL guidelines [26].

All patients undergo systematic and periodic screening for chronic diabetic complications in accordance with the schedules and modalities recommended by current guidelines. Macrovascular complications included acute myocardial infarction (AMI), chronic ischemic heart disease, heart failure, peripheral artery disease and stroke. Cardiac events were diagnosed via ECG, echocardiography, exercise stress testing or coronary CT angiography performed in the presence of atypical symptoms or baseline ECG abnormalities. Stroke was defined as a focal neurological deficit confirmed by neuroimaging. Peripheral artery disease (PAD) was periodically assessed through ankle‐brachial index (ABI) measurement and/or colour‐doppler ultrasound in patients aged ≥ 65 years, those with long‐standing diabetes (≥ 10 years), or in the presence of suggestive symptoms or clinical signs [27]. Diabetic kidney disease was periodically screened by estimating the Glomerular Filtration Rate (eGFR) applying the 2021 CKD‐EPI formula and assessing micro‐ or macro‐albuminuria via urinary albumin‐to‐creatinine ratio (UACR) [28]. Diabetic retinopathy was screened annually by dilated direct ophthalmoscopy, while diabetic peripheral neuropathy was assessed yearly through comprehensive foot assessments (skin inspection and neurological testing, such as the 10‐g monofilament and vibration perception), with electromyography reserved for cases where clinical findings warranted further investigation [29].

Written informed consent was obtained from all patients, and the Institutional Review Board of the district of Messina approved the study (protocol number 2016/85).

### 2.5 Statistical Analysis

Numerical data were expressed as median and interquartile range (first and third quartiles), and categorical variables as number and percentage. The non‐parametric approach was used because most of the numerical variables were not normally distributed, as verified by the Kolmogorov–Smirnov test. Then, the Wilcoxon Mann Whitney test was applied to compare numerical variables relative to glucometabolic balance and liver disease between the two follow‐up time points, before DAAs therapy and at the last evaluation in our outpatients, to highlight all the significant changes during this period. The same analysis was performed through the Chi Square Test for the categorical variables. Therefore, univariate and multivariate regression analyses were performed to identify variables associated with the main study outcomes: liver disease progression (defined as the occurrence of liver decompensation and/or hepatocellular carcinoma), T2D vascular complications (including both micro‐ and macro‐vascular events), and general progression, a composite endpoint encompassing both liver disease progression and the development of T2D vascular complications, as well as mortality. Finally, Kaplan–Meier curves were used to analyse the differences in HCC and T2D vascular complications' onset between cirrhotic and non‐cirrhotic patients. A sub‐analysis was performed on a group of patients to evaluate the presence of SNPs and their impact on the considered outcomes, applying univariate and multivariate logistic regression analysis. Statistical analyses were performed using SPSS 25.0 for Windows package. A P value lower than 0.05 has been considered statistically significant.

## 3 Results

### 3.1 Baseline Characteristics of Patients

One hundred‐eighty‐three T2D patients, treated with DAAs between April 1st, 2015, and December 31st, 2018, who attended the Medicine and Hepatology Unit and the Diabetes Unit until September 30th, 2024, have been prospectively enrolled in the study. The median follow‐up duration was 48 months (range 24–84), and 49 out of 183 (26.8%) patients completed the entire 108 months follow‐up period at our clinics. Demographic, biochemical, instrumental and clinical variables were collected at baseline, as summarized in Table 1. The cohort included 95 males (51.9%) and the median age was 67 years. At baseline, 81 patients (44.3%) had HCV‐related chronic liver disease, while 102 patients (55.7%) had compensated liver cirrhosis. Among the 102 cirrhotic patients, 11 (10.78%) had a history of hepatic decompensation before enrolment, and 40 (39.21%) presented gastric or oesophageal varices [33/102 (32.35%) with grade F1, 4/102 (3.92%) with F2, 1/102 (0.98%) with F3 and 2/102 (1.96%) with gastric varices, respectively]. At the beginning of the follow‐up, median liver stiffness measurement was 17.2 kPa (range 10.72–26 kPa), aminotransferase levels were over the upper normal limit with a median of 50 U/L (range 35–79.5 U/L) for AST and 63 U/L (range 37.5–93 U/L) for ALT. Also, 78 patients (42.6%) had previously received interferon‐based therapies without achieving a sustained virological response. Regarding metabolic parameters, study population had a median BMI of 25.95 kg/m^2^ (range 23.69–28.87 kg/m^2^), a median serum HbA1c of 7% (range 6.27%–7.9%), a median LDL‐c of 87.4 mg/dL (range 69.4–113.4 mg/dL) and a median TyG index of 4.67 (range 4.54–4.90). Focusing on T2D treatment, 23 patients (12.6%) were on nutritional therapy, 67 (36.6%) were treated with insulin, 79 (43.2%) received oral hypoglycaemic drugs and 14 (7.7%) assumed both. Lipid‐lowering therapies were prescribed to 28 patients (15.3%). Prior to DAAs initiation, 65 patients (35.5%) exhibited macrovascular T2D complications [18/183 (9.8%) had experienced major cardiovascular events (MACE), 28/183 (15.3%) had peripheral artery disease (PAD) and 19/183 (10.38%) had both], while 43 (23.5%) showed microvascular T2D complications [26/183 had nephropathy (14.2%), 13/183 (7.1%) had neuropathy and 10/182 (5.5%) retinopathy, either isolated or in combination], and among them 20 (10.92%) experienced both.

**TABLE 1:** Demographic, biochemical, clinical and instrumental data of 183 patients at baseline. All the categorical variables are expressed as number and percentage; all the numerical variables are expressed as median and interquartile range (first and third quartiles).

Variables	Baseline
Male gender, *n*	95 (51.9)
Age, years	67 (60–73)
Median follow‐up duration, months	48 (24–84)
Liver cirrhosis, *n*	102 (55.7)
Previous liver decompensation, *n*	11 (6)
BMI, kg/m^2^	25.95 (23.69–28.87)
Liver stiffness, kPa	17.2 (10.72–26)
Albumin, g/dL	3.9 (3.61–4.10)
Gamma‐globulins, g/dL	1.53 (1.26–1.73)
Creatinine, mg/dL	0.90 (0.70–1.00)
HbA1c, %	7 (6.27–7.9)
Serum fasting glucose, mg/dL	130 (108–151)
TyG index	4.67 (4.54–4.9)
Total cholesterol, mg/dL	157 (138–183)
HDL‐c, mg/dL	48 (37–58)
LDL‐c, mg/dL	87.4 (69.4–113.4)
Triglycerides, mg/dL	95 (76–124)
AST, U/L	50 (35–79.5)
ALT, U/L	63 (37.5–93)
GGT, U/L	58 (30.5–106)
Total bilirubin, mg/dL	0.74 (0.58–1)
Portal Vein diameter, mm	11.5 (10.2–12.5)
Interpolar Spleen diameter, cm	12 (10.6–14)
HCV RNA IU/mL	1 380 000 (518000–3 285 000)
HCV genotype, *n*	
1a	21 (11.47)
1b	108 (59)
2	38 (20.7)
3	5 (2.73)
4	11 (6)
Varices, grade	
F1	33 (18)
F2	4 (2.2)
F3	1 (0.5)
Gastric	2 (1.1)
Previous HCV therapy, *n*	78 (42.6)
Microvascular complications, *n*	43 (23.5)
Macrovascular complications, *n*	65 (35.5)
Insulin therapy, *n*	67 (36.6)
Oral Hypoglycaemic therapies, *n*	79 (43.2)
Both, *n*	14 (7.7)
Lipid lowering therapies, *n*	28 (15.3)
Non‐selective beta‐blockers, *n*	21 (11.47)

Abbreviations: ALT, alanine aminotransferase; AST, aspartate aminotransferase; BMI, Body Mass Index; GGT, gamma glutamyl transpeptidase; HbA1c, Glycated haemoglobin; HCV, Hepatitis C virus; HDL‐c, high‐density lipoprotein cholesterol; LDL‐c, low‐density lipoprotein cholesterol; TyG index, Triglyceride‐Glucose index.

### 3.2 Clinical Outcomes After DAAs Therapy

**FIGURE 1:**
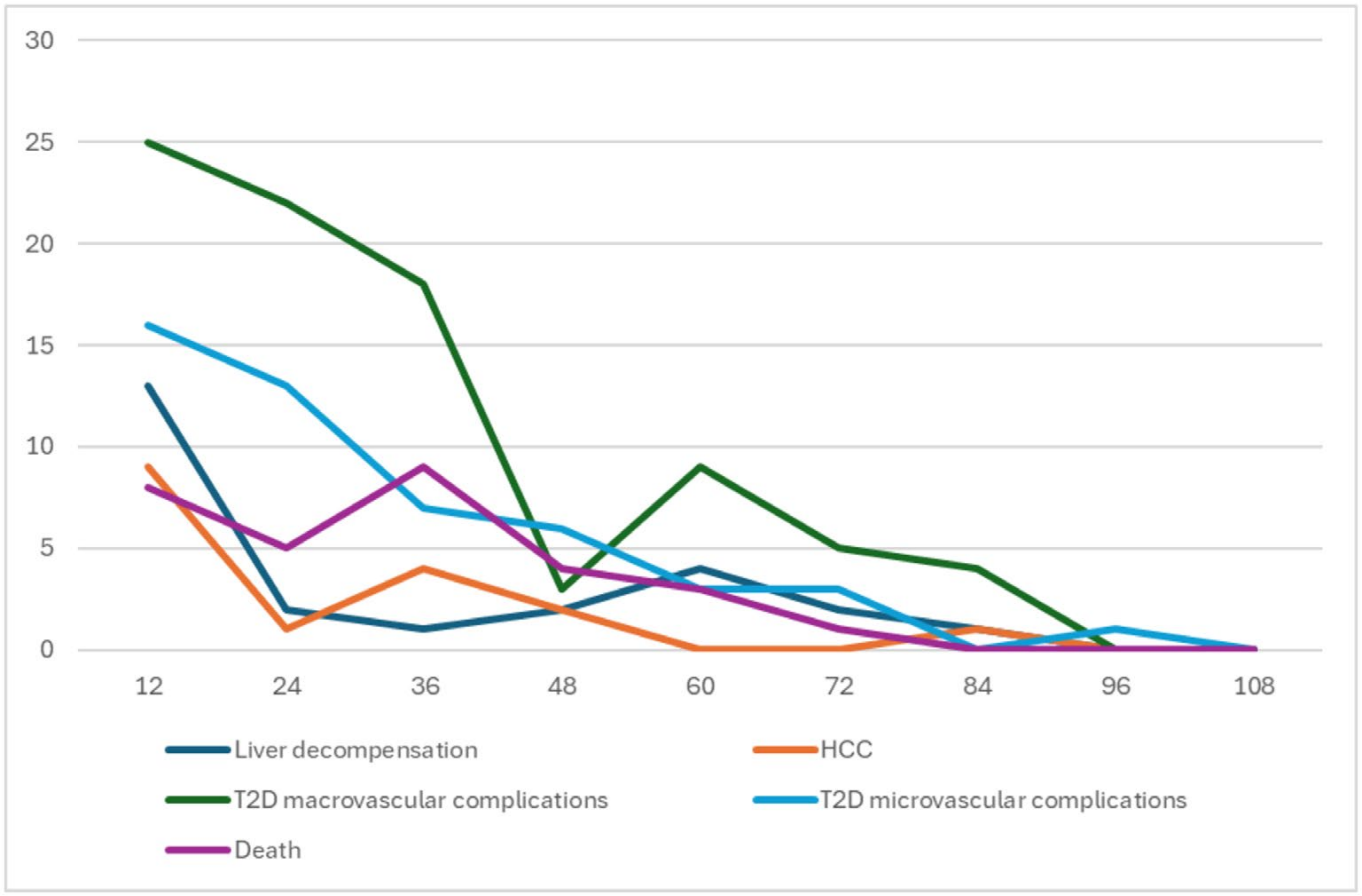
Graphical representation of clinical outcomes (coloured lines) occurrence after SVR (time 0 in the graphics). The x‐axis represents time in months, and the y‐axis represents the absolute number of patients experiencing the event (clinical outcomes) during the follow‐up period. HCC, hepatocellular carcinoma; SVR Sustained Virological Response; T2D, Type 2 diabetes.

Following treatment with direct‐acting antivirals, all enrolled patients achieved sustained virological response. Based on the considered clinical outcomes, 50 of 183 patients (27.3%) maintained a clinical stability over time for both T2D and liver disease, while 32 of 183 patients (17.48%) died, 15 of them (15/32, 46.8%) due to the progression of liver disease (Table S1). Regarding liver‐related outcomes, 38/183 patients (20.8%) experienced disease progression, defined as liver decompensation (19/183, 10.38%), hepatocellular carcinoma onset (10/183, 5.46%) or both (9/183, 4.9%). Additionally, three patients (1.6%) underwent orthotopic liver transplantation. Analysis of the timing of liver‐related complications revealed that liver decompensation predominantly occurred within the first year after SVR [13 of 28 patients (46%)], with a similar trend observed for HCC onset [9 out of 19 patients (47%) in the first year after SVR]. Vascular complications occurred in 93 of 183 patients (50.8%). Among the overall cohort, major cardiovascular events occurred in 11/183 patients (6%), peripheral artery disease in 66/183 (36%), and both conditions in 16/183 (8.7%). Moreover, 62/183 patients (33.9%) developed new microvascular complications: nephropathy (38/183, 20.8%), neuropathy (21/183, 11.5%), retinopathy (13/183, 7.1%), either isolated or in combination. Temporal analysis showed that macrovascular complications occurred mainly within the first 2 years following SVR [25 out of 93 (26.88%) in the first year; 22 of 93 (23.65%) in the second], with a similar distribution observed for microvascular complications [16 of 62 (25.8%) in the first year, 13 of 62 (20.9%) in the second] (Figure 1). Thus, the Wilcoxon Mann–Whitney Test has been applied to compare continuous numerical variables between baseline and the last outpatient evaluation. This analysis showed a significant reduction in liver stiffness measurement (*p* < 0.001), serum gamma‐globulins (*p* < 0.001), HbA1c (*p* = 0.006), and aminotransferase levels (respectively AST, ALT, and GGT with a *p* < 0.001). Conversely, both BMI (*p* = 0.015) and serum creatinine levels (*p* < 0.001) significantly increased during the follow‐up period (Table 2). Chi‐Square test was applied to analyse categorical variables, revealing a significant increase in the incidence of both macrovascular (*p* = 0.003) and microvascular (*p* = 0.02) T2D complications, as well as in the use of lipid‐lowering therapies (*p* < 0.001) (Table 2).

**TABLE 2:** Demographic, biochemical, clinical and instrumental data of 183 patients, comparing data at baseline and at the last evaluation in our outpatient. All the categorical variables are expressed as number and percentage; all the numerical variables are expressed as median and interquartile range (first and third quartiles).

Variables	Baseline	Last follow‐up	*P*
BMI, kg/m^2^	25.95 (23.69–28.87)	27.3 (23.9–30.47)	**0.015**
Liver stiffness, kPa	17.2 (10.72–26)	8.4 (5.72–13.72)	**< 0.001**
Albumin, g/dL	3.9 (3.61–4.10)	4 (3.6–4.26)	0.530
Gamma‐globulins, g/dL	1.53 (1.26–1.73)	1.19 (1.01–1.44)	**< 0.001**
Creatinine, mg/dL	0.90 (0.70–1.00)	0.90 (0.80–1.20)	**< 0.001**
HbA1c, %	7 (6.27–7.9)	6.7 (6.1–7.6)	**0.006**
Serum fasting glucose, mg/dL	130 (108–151)	129 (109–158)	0.247
TyG index	4.67 (4.54–4.90)	4.76 (4.58–4.94)	0.627
Total cholesterol, mg/dL	157 (138–183)	165 (135–189)	0.497
HDL‐c, mg/dL	48 (37–58)	47 (40–57)	0.896
LDL‐c, mg/dL	87.4 (69.4–113.4)	90.8 (70–113.8)	0.802
Triglycerides, mg/dL	95 (76–124)	99 (75.5–134)	0.291
AST, U/L	50 (35–79.5)	22 (17–28)	**< 0.001**
ALT, U/L	63 (37.5–93)	20 (14–29)	**< 0.001**
GGT, U/L	58 (30.5–106)	23 (17–42)	**< 0.001**
Total bilirubin, mg/dL	0.74 (0.58–1)	0.69 (0.50–1.00)	0.169
Microvascular complications, *n*	43 (23.5)	62 (33.9)	**0.02**
Macrovascular complications, *n*	65 (35.5)	93 (50.8)	**0.003**
Insulin therapy, *n*	67 (36.6)	61 (33.3)	0.51
Oral Hypoglycemic therapies, *n*	79 (43.2)	69 (37.7)	0.28
Both, *n*	14 (7.7)	19 (10.4)	0.36
Lipid lowering therapies, *n*	28 (15.3)	74 (40.4)	**< 0.001**
Non‐selective beta‐blockers, *n*	21 (11.47)	24 (13.11)	0.63

*Note:* The statistically significant results are highlighted in bold in the table.

Abbreviations: ALT, alanine aminotransferase; AST, aspartate aminotransferase; BMI, Body Mass Index; GGT, gamma glutamyl transpeptidase; HbA1c, Glycated haemoglobin; HDL‐c, high‐density lipoprotein cholesterol; LDL‐c, low‐density lipoprotein cholesterol; TyG index, Triglyceride‐Glucose index.

### 3.3 Independent Predictors of Liver and Metabolic‐Related Outcomes

Univariate and multivariate regression analyses were conducted to identify baseline variables that were independently associated with the selected outcomes at the end of the follow‐up period. When considering a composite outcome defined as *general progression*—including both liver disease progression and T2D vascular complications onset—univariate regression analysis identified a significant association with higher baseline TyG index values (OR 1.649, 95% C.I. 1.025–2.653, *p* = 0.039), higher AST (OR 1.010, 95% C.I. 1.000–1.021, *p* = 0.045) and GGT (OR 1.005, 95% C.I. 1.000–1.011, *p* = 0.047) levels. At multivariate regression analysis, TyG index emerged as the only independent predictive factor (OR 1.647, 95% C.I. 1.029–2.637, *p* = 0.038) of general progression (Table 3).

**TABLE 3:** Univariate and multivariate regression analysis for the composite outcome of liver disease progression and T2D vascular complications onset (general progression).

General progression	Univariate model			Multivariate model		
Variables	OR	95% C.I.	*p*	OR	95% C.I.	*p*
Age, years	1.025	0.991–1.060	0.154	—	—	—
Male gender	1.241	0.647–2.379	0.516	—	—	—
Liver cirrhosis	1.231	0.641–2.363	0.533	—	—	—
BMI, kg/m^2^	0.983	0.916–1.056	0.644	—	—	—
Liver stiffness, kPa	1.021	0.992–1.051	0.157	—	—	—
Albumin, g/dL	0.694	0.295–1.632	0.402	—	—	—
Gamma‐globulins, g/dL	1.027	0.543–1.943	0.935	—	—	—
Creatinine, mg/dL	1.403	0.381–5.158	0.611	—	—	—
HbA1c, %	1.205	0.917–1.584	0.180	—	—	—
Serum fasting glucose, mg/dL	1.005	0.996–1.014	0.267	—	—	—
TyG index	1.649	1.025–2.653	0.039	1.647	1.029–2.637	**0.038**
Total cholesterol, mg/dL	0.991	0.981–1.002	0.106	—	—	—
Triglycerides, mg/dL	1.003	0.997–1.010	0.296	—	—	—
LDL‐c, mg/dL	0.991	0.979–1.003	0.151	—	—	—
ALT, U/L	1.004	0.998–1.011	0.215	—	—	—
AST, U/L	1.010	1.000–1.021	0.045	1.009	0.997–1.021	0.129
GGT, U/L	1.005	1.000–1.011	0.047	1.003	0.997–1.009	0.272
Total bilirubin, mg/dL	0.904	0.537–1.522	0.704	—	—	—
Previous decompensation	1.756	0.366–8.425	0.482	—	—	—
Portal Vein diameter, mm	1.189	0.938–1.507	0.153	—	—	—
Spleen diameter, cm	0.961	0.910–1.015	0.152	—	—	—
Varices	0.871	0.411–1.847	0.782	—	—	—

*Note:* The statistically significant results are highlighted in bold in the table.

Abbreviations: ALT, alanine aminotransferase; AST, aspartate aminotransferase; BMI, Body Mass Index; GGT, gamma glutamyl transpeptidase; HbA1c, Glycated haemoglobin; HDL‐c, high‐density lipoprotein cholesterol; LDL‐c, low‐density lipoprotein cholesterol; TyG index, Triglyceride‐Glucose index.

Liver disease progression—defined as the occurrence of hepatic decompensation and/or hepatocellular carcinoma—was significantly related at univariate analysis to presence of liver cirrhosis prior to therapy (OR 21.545, 95% C.I. 4.999–92.859, *p* < 0.001), higher liver stiffness values (OR 1.072, 95% C.I. 1.040–1.105, *p* < 0.001), elevated serum gamma‐globulins (OR 2.024, 95% C.I. 1.046–3.918, *p* = 0.036), AST (OR 1.008, 95% C.I. 1.001–1.016, *p* = 0.033) and bilirubin (OR 3.752, 95% C.I. 1.858–7.578, *p* < 0.001) levels, increased portal vein diameter (OR 1.626, 95% C.I. 1.238–2.135, *p* < 0.001), and history of previous hepatic decompensation (OR 22.034, 95% C.I. 4.523–107.338, *p* < 0.001). The presence of oesophageal varices was inversely associated with liver disease progression (OR 0.122, 95% C.I. 0.272–0.55, *p* < 0.001). In multivariate regression analysis, previous decompensation (OR 20.535, 95% C.I. 2.312–182.392, *p* = 0.007) and baseline liver cirrhosis (OR 8.805, 95% C.I. 1.396–55.525, *p* = 0.021) remained independently associated with liver disease progression. Additionally, the presence of varices (OR 0.326, 95% C.I. 0.119–0.892, *p* = 0.029) was associated with a lower risk of liver‐related events (Table 4).

**TABLE 4:** Univariate and multivariate regression analysis for the composite outcome of liver decompensation and hepatocellular carcinoma onset (liver disease progression).

Liver disease progression	Univariate model			Multivariate model		
Variables	OR	95% C.I.	*p*	OR	95% C.I.	*p*
Age, years	1.038	0.998–1.079	0.064	—	—	—
Male gender	1.787	0.857–3.727	0.122	—	—	—
Liver cirrhosis	21.545	4.999–92.859	< 0.001	8.805	1.396–55.525	**0.021**
BMI, kg/m^2^	1.006	0.931–1.087	0.877	—	—	—
Liver stiffness, kPa	1.072	1.040–1.105	< 0.001	1.035	0.995–1.076	0.085
Albumin, g/dL	0.438	0.172–1.120	0.085	—	—	—
Gamma‐globulins, g/dL	2.024	1.046–3.918	0.036	1.325	0.546–3.216	0.533
Creatinine, mg/dL	1.211	0.306–4.796	0.785	—	—	—
HbA1c, %	1.144	0.883–1.483	0.308	—	—	—
Serum fasting glucose, mg/dL	1.000	0.992–1.009	0.973	—	—	—
TyG index	1.190	0.672–2.108	0.551	—	—	—
Total cholesterol, mg/dL	0.996	0.985–1.007	0.447	—	—	—
Triglycerides, mg/dL	1.000	0.994–1.006	0.954	—	—	—
LDL‐c, mg/dL	0.987	0.974–1.001	0.071	—	—	—
ALT, U/L	1.000	0.994–1.006	0.971	—	—	—
AST, U/L	1.008	1.001–1.016	0.033	0.999	0.987–1.011	0.849
GGT, U/L	1.002	0.999–1.006	0.227	—	—	—
Total bilirubin, mg/dL	3.752	1.858–7.578	< 0.001	2.532	0.904–7.089	0.077
Previous decompensation	22.034	4.523–107.338	< 0.001	20.535	2.312–182.392	**0.007**
Portal Vein diameter, mm	1.626	1.238–2.135	< 0.001	0.894	0.606–1.319	0.572
Spleen diameter, cm	1.003	0.970–1.038	0.848	—	—	—
Varices	0.122	0.055–0.272	< 0.001	0.326	0.119–0.892	**0.029**

*Note:* The statistically significant results are highlighted in bold in the table.

Abbreviations: ALT, alanine aminotransferase; AST, aspartate aminotransferase; BMI, Body Mass Index; GGT, gamma glutamyl transpeptidase; HbA1c, Glycated haemoglobin; LDL‐c, low‐density lipoprotein cholesterol; TyG index, Triglyceride‐Glucose index.

Vascular T2D complications—including both microvascular and macrovascular complications—were analysed as a composite outcome. In the univariate regression analysis, lower levels of total cholesterol (OR 0.998, 95% C.I. 0.977–0.998, *p* = 0.017) and LDL‐c (OR 0.985, 95% C.I. 0.973–0.997, *p* = 0.017) were associated with the onset of vascular complications. Neither variable remained significant in the multivariate regression analysis (total cholesterol OR 1.002, 95% C.I. 0.982–1.023, *p* = 0.815; LDL‐c OR 0.983, C.I. 0.962–1.005, *p* = 0.131).

Regression analyses were also conducted to identify factors associated with mortality. In univariate regression analysis, higher serum LDL‐c (OR 1.022, 95% C.I. 1.011–1.044, *p* = 0.038), ALT (OR 1.009, 95% C.I. 1.001–1.017, *p* = 0.020), and bilirubin (OR 1.995, 95% C.I. 1.002–3.970, *p* = 0.049) levels were significantly associated with death occurrence. Among these, only elevated LDL‐c levels remained independently associated with mortality at multivariate regression analysis (OR 1.027, 95% C.I. 1.000–1.054, *p* = 0.048) (Table 5).

**TABLE 5:** Univariate and multivariate regression analysis for mortality.

Mortality	Univariate model			Multivariate model		
Variables	OR	95% C.I.	*p*	OR	95% C.I.	*p*
Age, years	1.036	0.966–1.111	0.320	—	—	—
Male gender	0.922	0.258–3.300	0.901	—	—	—
Liver cirrhosis	1.203	0.328–4.415	0.780	—	—	—
BMI, kg/m^2^	0.927	0.814–1.054	0.248	—	—	—
Liver stiffness, kPa	0.988	0.932–1.048	0.691	—	—	—
Albumin, g/dL	1.591	0.300–8.445	0.586	—	—	—
Gamma‐globulins, g/dL	1.152	0.347–3.820	0.817	—	—	—
Creatinine, mg/dL	0.257	0.012–5.459	0.384	—	—	—
HbA1c, %	0.822	0.470–1.437	0.491	—	—	—
Serum fasting glucose, mg/dL	0.998	0.981–1.015	0.818	—	—	—
TyG index	1.150	0.339–3.909	0.822	—	—	—
Total cholesterol, mg/dL	1.016	0.997–1.035	0.096	—	—	—
Triglycerides, mg/dL	1.000	0.989–1.011	0.973	—	—	—
LDL‐c, mg/dL	1.022	1.011–1.044	0.038	1.027	1.000–1.054	**0.048**
ALT, U/L	1.009	1.001–1.017	0.020	1.009	1.000–1.018	0.057
AST, U/L	1.009	0.998–1.020	0.096	—	—	—
GGT, U/L	1.002	0.996–1.009	0.440	—	—	—
Total bilirubin, mg/dL	1.995	1.002–3.970	0.049	0.985	0.252–3.852	0.983
Previous decompensation	1.800	0.207–15.646	0.594	—	—	—
Portal Vein diameter, mm	0.970	0.621–1.516	0.894	—	—	—
Spleen diameter, cm	0.904	0.705–1.159	0.427	—	—	—
Varices	2.619	0.322–23.316	0.368	—	—	—

*Note:* The statistically significant results are highlighted in bold in the table.

Abbreviations: ALT, alanine aminotransferase; AST, aspartate aminotransferase; BMI, Body Mass Index; GGT, gamma glutamyl transpeptidase; HbA1c, Glycated haemoglobin; LDL‐c, low‐density lipoprotein cholesterol; TyG index, Triglyceride‐Glucose index.

**FIGURE 2:**
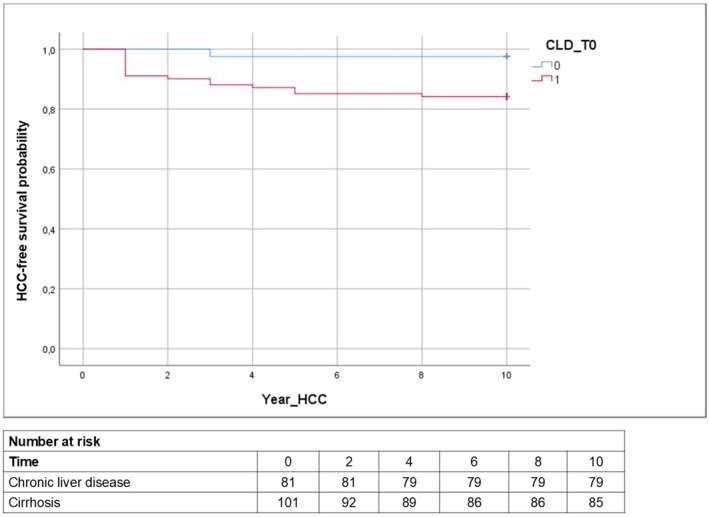
Kaplan–Meier analysis of HCC‐free survival according to baseline liver disease severity. Patients were stratified into chronic liver disease (0) and liver cirrhosis (1). Tick marks indicate censored observations. Numbers at risk at each follow‐up time point are shown below the x‐axis. Group differences were assessed using the log‐rank test (Log Rank test: *P* = 0.003). HCC, hepatocellular carcinoma.

Finally, Kaplan–Meier analyses were performed to compare cirrhotic and non‐cirrhotic patients for the considered outcomes. A statistically significant difference was observed only for the development of hepatocellular carcinoma, which was more frequent among patients with liver cirrhosis (Log Rank test: *p* = 0.003) (Figure 2).

### 3.4 Distribution of SNPs and Association With Liver and Metabolic‐Related Outcomes

Seventy‐eight out of 183 patients (42.6%) underwent blood sample collection for SNPs analysis. Baseline demographic, clinical, and biochemical characteristics in the genotyped subset were comparable to those of the overall cohort (Table S2). Genotype distributions were as follows: *PNPLA3* rs738409 (p.I148M), CC/CG/GG = 46 (58.97%), 24 (30.76%), 8 (10.25%); *TM6SF2* rs58542926 (p.E167K), CC/CT/TT = 71 (91.02%), 6 (7.69%), 1 (1.28%); and rs641738 at the *MBOAT7/TMC4* locus (commonly annotated *TMC4* p.G17E), CC/CT/TT = 19 (24.35%), 40 (51.20%), 19 (24.35%). The comparison of all the features collected before DAAs therapy and at the last evaluation in our outpatients showed a significant amelioration in liver stiffness (*p* < 0.001), gamma‐globulins (*p* < 0.001), HbA1c (*p* = 0.008) and aminotransferase levels (*p* < 0.001), and an increase in lipid lowering therapies consumption (*p* = 0.001), as previously highlighted in the whole study population (Table S3). Univariate and multivariate regression analyses were conducted using dominant genetic models (CG + GG vs. CC for *PNPLA3* rs738409; CT + TT vs. CC for *TM6SF2* rs58542926; CT + TT vs. CC for rs641738 at *MBOAT7/TMC4*). In univariate regression analysis, liver disease progression was related to higher liver stiffness values (OR 1.058, 95% C.I. 1.013–1.105, *p* = 0.011), higher total bilirubin (OR 8.667, 95% C.I. 2.332–32.215, *p* = 0.001) and portal vein diameter (OR 1.838, 95% C.I. 1.195–2.826, *p* = 0.006), lower serum albumin (OR 0.087, 95% C.I. 0.015–0.508, *p* = 0.007) and LDL‐c (OR 0.971, 95% C.I. 0.944–0.998, *p* = 0.037), presence of rs641738 CT + TT at *MBOAT7/TMC4* (OR 3.429, 95% C.I. 0.984–11.947, *p* = 0.050). At multivariate regression analysis, lower LDL‐c levels (OR 0.983, 95% C.I. 0.883–0.998, *p* = 0.041), higher total bilirubin values (OR 24.591, 95% C.I. 1.305–463.482, *p* = 0.033), and rs641738 CT + TT (OR 35.737, 95% C.I. 1.533–822.056, *p* = 0.025) remained independently associated with liver disease progression (Table S4). A non‐significant trend toward an increased risk of vascular complications has been noted for T2D, including vascular complication onset, death, and the overall progression outcome (Tables S5, S6, and S7, respectively).

## 4 Discussion

Chronic HCV infection represents a complex systemic disease. Although DAAs therapy achieves viral eradication, extrahepatic manifestations continue to influence patients' overall prognosis [30]. Within this context, the present clinical study provides a real‐world evaluation of the prognostic determinants of metabolic and hepatic outcomes in diabetic patients who obtained HCV cure following DAAs therapy. The long follow‐up period enabled a comprehensive assessment of both liver disease‐related complications and diabetes‐associated vasculopathy, underscoring the importance of early antiviral treatment and precise cardiometabolic risk management. The study also offers innovative insights by genotyping polymorphisms associated with metabolic liver disease, allowing us to investigate their contribution to long‐term disease progression.

Despite the metabolic and hepatic improvements observed after SVR, only a minority of patients maintained clinical stability overtime (27.3%). Alongside the improvement in liver stiffness (*p* < 0.001), the favourable changes in laboratory parameters related to hepatic function—such as gamma‐globulins (*p* < 0.001) and aminotransferases (*p* < 0.001) and the reduction in HbA1c levels (*p* = 0.006), our patients experienced a worsening of BMI (*p* = 0.015) and serum creatinine (*p* < 0.001), and an increase in both micro‐ and macrovascular complications' onset (*p* = 0.02, *p* = 0.003, respectively). Statistical analyses identified insulin resistance, expressed by the TyG index [31], as a determinant of both new‐onset T2D and liver‐related events, whereas elevated LDL‐c levels emerged as a long‐term predictor of mortality.

The intricate relationship between chronic HCV infection, metabolic dysfunction, and T2D is well established, as the virus contributes to the development of hepatic steatosis not only through direct mechanisms but also by promoting the onset of metabolic syndrome [7, 32]. Although our cohort exhibited an improvement in HbA1c levels, TyG index values remained persistently elevated through follow‐up, and the incidence of diabetes‐related micro‐ and macrovascular complications increased over time. Regarding the incidence of T2D vascular complications, nephropathy rates in our cohort were comparable to those reported in the general T2D population [33], whereas the incidence of retinopathy and neuropathy was lower [34, 35]. A direct comparison for PAD was not feasible due to the wide variability observed across studies [36]. These results reflect the complex and sometimes discordant metabolic changes observed after SVR. Previous studies have reported an increase in LDL‐c levels following SVR, which may contribute to the development of atherosclerosis‐related vascular complications [30], as viral replication seems to interfere with hepatic cholesterol synthesis. It can be hypothesized that HCV eradication influences lipidic homeostasis and plaque development [37], which could justify the high incidence of vascular complications in our cohort. Indeed, although LDL‐c levels did not increase during follow‐up in our population, the significant rise in lipid‐lowering therapy prescriptions (*p* < 0.001) may explain this stability. Consistent with this metabolic vulnerability, elevated LDL‐c levels were independently associated with mortality in the multivariate model, a datum that highlights once again the critical role of metabolic comorbidities in determining the overall prognosis of these patients.

When examining liver disease‐related outcomes more specifically, our cohort exhibited markedly lower rates of decompensation than those commonly reported in the broader cirrhotic population, suggesting a favourable effect of DAAs on liver disease natural history [23]. Nonetheless, 20.8% of patients experienced liver disease progression, primarily associated with baseline clinically significant portal hypertension [38]. This finding reinforces the relevance of early antiviral intervention and aligns with previous studies reporting improved liver function and fibrosis indices after SVR [39]. Importantly, the long‐term follow‐up in our study reveals that improvements in liver stiffness may persist well beyond the early post‐SVR period, possibly reflecting sustained reduction in hepatic inflammation and type III collagen deposition [30]. Interestingly, the presence of oesophageal varices appeared to exert a protective effect on liver disease progression, likely attributable not to the varices themselves but to the administration of non‐selective beta blockers, known to reduce portal hypertension and the consequent risk of liver decompensation [40].

Beyond the clinical and laboratory predictors, host genetic variation emerged as a relevant determinant of liver disease progression. Previous clinical studies have examined the impact of *PNPLA3* p.I148M (rs738409), *TM6SF2* p.E167K (rs58542926) and rs641738 at the *MBOAT7/TMC4* locus in patients with HCV and HIV coinfection on liver disease progression after achieving SVR [41, 42]. However, to our knowledge, the present study is the first to assess their long‐term impact in patients also presenting type 2 diabetes. The rs641738 variant at the *MBOAT7/TMC4* locus was highly prevalent in our cohort (T‐allele carriage 75.6%) and remained an independent predictor of liver disease progression after SVR (*p* = 0.025), suggesting its involvement in phospholipid remodelling and fibrogenic pathways [43]. Conversely, according to previous studies [44], no significant associations were observed between liver disease progression and *PNPLA3* or *TM6SF2* variants. Notably, rs641738 showed a nonsignificant trend toward higher rates of vascular complications, suggesting a predominantly hepatic rather than systemic metabolic effects [45].

In the broader evaluation of hepatological outcomes, the occurrence of HCC remains a central issue. It is well established that patients with chronic HCV infection and cirrhosis retain a substantial risk of HCC development for up to a decade after SVR, particularly in the presence of metabolic comorbidities [46]. This long‐term risk provides the rationale for current surveillance strategies, which recommend biannual alpha‐fetoprotein testing and ultrasound examinations [25, 47, 48, 49]. In our cohort, HCC was diagnosed predominantly during the first year after SVR 9/19 patients (47.3%), with a subsequent progressive decline in incidence during follow‐up, consistent with existing literature. Moreover, baseline cirrhosis was significantly associated with HCC onset, further reinforcing the importance of early antiviral treatment to reduce the burden of long‐term complications.

Taken together these findings suggests that metabolic dysfunction played a pivotal role in determining long‐term prognosis in this specific cohort, exerting substantial influence on both vascular complications and liver‐related events [50], also underscoring the need for careful management of metabolic risk factors even after successful viral eradication. Additionally, early HCV treatment has a substantial impact on liver disease progression, reinforcing the need for active screening programs within primary care to ensure timely therapy.

This study has several limitations, the most significant being the high rate of follow‐up discontinuation. Overall, only 49 (26.8%) and 28 patients (15.3%) completed the long‐term monitoring for liver disease and T2D, respectively. Follow‐up discontinuation was mainly related to referral of patients with liver stiffness values < 8 kPa and stable glycaemic control to general practitioners, as well as to the impact of the SARS‐CoV‐2 pandemic. Notably, the highest rates of follow‐up interruption occurred between 2019 and 2020. Therefore, unrecorded deaths or progression in liver disease and T2D may have occurred, potentially limiting the interpretation of the study results.

Moreover, antidiabetic therapies were only partially explored. Novel agents introduced in recent years, such as sodium‐glucose co‐transporter 2 inhibitors (SGLT2i) and glucagon‐like peptide‐1 receptor agonists (GLP1ra), could not be adequately assessed due to heterogeneous therapeutic modifications, making analytical standardization challenging. Additionally, the potential effects of non‐selective beta blockers on liver disease progression were not evaluated, representing another limitation of the study. Finally, the limited number of patients who underwent genetic testing reduces the statistical power of this subgroup analysis: a larger population would allow a more precise characterization of the genetic profile and a deeper understanding of the role of these polymorphisms in metabolic and liver disease progression.

## 5 Conclusions

Hepatitis C virus infection treatment with DAAs significantly improves liver disease natural history, reducing decompensation rates. The present study confirms the importance of early treatment, as the severity of hepatic disease before initiating DAAs therapy notably influences liver‐related outcomes. Furthermore, active surveillance for hepatocellular carcinoma is warranted, along with rigorous management of metabolic risk factors that strongly contribute to liver disease progression. Beyond clinical factors, the rs641738 variant at the *MBOAT7/TMC4* locus emerged as an independent predictor of disease progression following SVR in a limited number of subgroups of patients. This finding suggests a potential role of genetic information in patient risk stratification, potentially paving the way for tailored surveillance and therapeutic strategies.

## Author Contributions

Clelia Asero: investigation, data curation, formal analysis, resources, writing original draft; Maria Stella Franzè: visualization, formal analysis, supervision, writing review and editing; Teresa Maltese: data curation, resources; Alberto La Spada: data curation, resources; Daniele Lombardo: methodology, resources; Claudia Grisanti: data curation, investigation; Giuseppina Russo: visualization, validation, writing review and editing; Annalisa Giandalia: visualization, validation, writing review and editing; Concetta Pitrone: data curation, visualization; Roberto Filomia: data curation, visualization; Gaia Caccamo: data curation, visualization; Carlo Saitta: data curation, visualization; Anna Licata: visualization, writing review and editing; Teresa Pollicino: conceptualization, supervision, visualization, methodology, validation, writing review and editing; Irene Cacciola: conceptualization, supervision, visualization, methodology, validation, writing review and editing.

## Funding

‘Multidisciplinary Network for the screening, diagnosis and treatment of Advanced Chronic Liver Disease (ACLD): an integrated and advanced management model’. CUP J53D23018010001—ID PRIN_2022PNRR_P2022KCHLN_002 and PNRR‐MAD‐2022‐12 375 656.

## Conflicts of Interest

The authors declare no conflicts of interest.

## Supporting information


**Table S1:** Causes and timing of mortality during follow‐up after sustained virological response (SVR). All the variables are expressed as number and percentage.
**Table S2:** Demographic, biochemical, clinical and instrumental data of 78 patients underwent genetic analysis at baseline. All the categorical variables are expressed as number and percentage; all the numerical variables are expressed as median and interquartile range (first and third quartiles).
**Table S3:** Demographic, biochemical, clinical and instrumental data of 78 patients underwent genetic analysis, comparing data at baseline and at the last evaluation in our outpatient. All the categorical variables are expressed as number and percentage; all the numerical variables are expressed as median and interquartile range (first and third quartiles).
**Table S4:** Univariate and multivariate regression analysis for the composite outcome of liver decompensation and hepatocellular carcinoma onset (liver disease progression).
**Table S5:** Univariate and multivariate regression analysis for the composite outcome of T2D vascular complications (micro‐and macro‐vascular complications).
**Table S6:** Univariate and multivariate regression analysis for mortality.
**Table S7:** Univariate and multivariate regression analysis for the composite outcome of general progression (T2D vascular complications and liver disease progression).

## Data Availability

The data that support the findings of this study are available from the corresponding author upon reasonable request.
