# Supplementary material for: From Cure to Complexity: Post‐SVR Liver and Metabolic Trajectories in Diabetic Patients
Source: Liver Int. 2026 Apr 17;46(5):e70631. doi: 10.1111/liv.70631 (PMC13090614; doi:10.1111/liv.70631)
Supplement: Supplementary file 1 — Table S1: Causes and timing of mortality during follow‐up after sustained virological response (SVR). All the variables are expressed as number and percentage. Table S2: Demographic, biochemical, clinical and instrumental data of 78 patients underwent genetic analysis at baseline. All the categorical variables are expressed as number and percentage; all the numerical variables are expressed as median and interquartile range (first and third quartiles). Table S3: Demographic, biochemical, clinical and instrumental data of 78 patients underwent genetic analysis, comparing data at baseline and at the last evaluation in our outpatient. All the categorical variables are expressed as number and percentage; all the numerical variables are expressed as median and interquartile range (first and third quartiles). Table S4: Univariate and multivariate regression analysis for the composite outcome of liver decompensation and hepatocellular carcinoma onset (liver disease progression). Table S5: Univariate and multivariate regression analysis for the composite outcome of T2D vascular complications (micro‐and macro‐vascular complications). Table S6: Univariate and multivariate regression analysis for mortality. Table S7: Univariate and multivariate regression analysis for the composite outcome of general progression (T2D vascular complications and liver disease progression). [file LIV-46-0-s001.docx]

**Supplementary Table S1.** Causes and timing of mortality during follow-up after sustained virological response (SVR). All the variables are expressed as number and percentage.

| **Variables** | **Death (n = 32)** |
| --- | --- |
| **Cause of death, n**  Liver disease progression  Major cardiovascular events  Other causes | 15 (46.8)  5 (15.6)  12 (37.5) |
| **Timing of death after SVR, months**  Twelve  Twenty-four  Thirty-six  Forty-eight  Sixty  Seventy-two | 8 (25)  5 (15.6)  9 (28.1)  6 (18.7)  3 (9.3)  1 (3.1) |

*Abbreviation: SVR (Sustained Virological Response).*

**Supplementary Table S2.** Demographic, biochemical, clinical and instrumental data of 78 patients underwent genetic analysis at baseline. All the categorical variables are expressed as number and percentage; all the numerical variables are expressed as median and interquartile range (first and third quartiles).

| **Variables** | **Baseline** |
| --- | --- |
| Male gender, n | 40 (51.3) |
| Age, years | 68 (61 – 71) |
| Median follow-up duration, months | 60 (36 – 84) |
| Liver cirrhosis, n | 40 (51.3) |
| Previous liver decompensation, n | 5 (6.4) |
| BMI, kg/m^2^ | 26.93 (24.44 – 29.68) |
| Liver stiffness, kPa | 14.3 (10 – 26) |
| Albumin, g/dL | 3.89 (3.6 – 4.1) |
| Gamma-globulins, g/dL | 1.52 (1.21 – 1.75) |
| Creatinine, mg/dL | 0.9 (0.7 – 1.1) |
| HbA1c, % | 7.1 (6.3 – 7.9) |
| Serum fasting glucose, mg/dL | 127 (108 – 150) |
| TyG index | 4.65 (4.52 – 4.9) |
| Total cholesterol, mg/dL | 164 (146.25 – 188) |
| HDL-c, mg/dL | 48.5 (35 – 58.25) |
| LDL-c, mg/dL | 85.4 (70.30 – 118.25) |
| Triglycerides, mg/dL | 96 (72.5 – 134.5) |
| AST, U/L | 50 (32 – 83.5) |
| ALT, U/L | 60 (35.75 – 87.25) |
| GGT, U/L | 44.5 (29 – 103.5) |
| Total bilirubin, mg/dL | 0.73 (0.60 – 1.00) |
| Portal Vein diameter, mm | 11 (10 – 12) |
| Interpolar Spleen diameter, cm | 11.7 (10.3 – 14) |
| HCV RNA IU/mL | 1310000 (474000 – 2828787.50) |
| HCV genotype  *1a*  *1b*  *2*  *3*  *4* | 10 (12.8)  42 (53.84)  21 (26.92)  1 (1.3)  4 (5.1) |
| Varices, grade  F1  F2  F3  gastric | 12 (15.4)  2 (2.6)  0 (0)  1 (1.3) |
| Previous HCV therapy, n | 30 (38.5) |
| Microvascular complications, n | 21 (26.9) |
| Macrovascular complications, n | 35 (44.9) |
| Insulin therapy, n | 28 (35.9) |
| Oral Hypoglycemic therapies, n | 35 (44.9) |
| Both, n | 6 (7.7) |
| Lipid lowering therapies, n | 19 (24.4) |
| PNPLA3 rs738409 I148 (CC/CG/GG), n | 46 (58.97) /24 (30.76) /8 (10.25) |
| TM6SF2 rs58542926 E167K (CC/CT/TT), n | 71 (91.02) / 6 (7.69) /1 (1.28) |
| MBOAT7 (TMC4) rs641738 G17E (CC/CT/TT), n | 19 (24.35) / 40 (51.2) / 19 (24.35). |

*Abbreviations: BMI (Body Mass Index), HbA1c (Glycated haemoglobin), TyG index (Triglyceride-Glucose index), HDL-c (high-density lipoprotein cholesterol), LDL-c (low-density lipoprotein cholesterol), AST (aspartate aminotransferase), ALT (alanine aminotransferase), GGT (gamma glutamyl transpeptidase), HCV (Hepatitis C virus), PNPLA3 (Patatin-like phospholipase domain-containing protein 3), TM6SF2 (Transmembrane 6 Superfamily Member 2), MBOAT7 (Membrane-bound O-acyltransferase 7)*.

**Supplementary Table S3.** Demographic, biochemical, clinical and instrumental data of 78 patients underwent genetic analysis, comparing data at baseline and at the last evaluation in our outpatient. All the categorical variables are expressed as number and percentage; all the numerical variables are expressed as median and interquartile range (first and third quartiles).

| **Variables** | **Baseline** | **Last follow-up** | **P value** |
| --- | --- | --- | --- |
| BMI, kg/m^2^ | 26.93 (24.44 – 29.68) | 27.3 (24.2 – 30.5) | 0.842 |
| Liver stiffness, kPa | 14.3 (10 – 26) | 7.05 (5.05 – 11.92) | **< 0.001** |
| Albumin, g/dL | 3.89 (3.6 – 4.1) | 3.82 (3.58 – 4.17) | 0.988 |
| Gamma-globulins, g/dL | 1.52 (1.21 – 1.75) | 1.17 (0.95 – 1.34) | **< 0.001** |
| Creatinine, mg/dL | 0.9 (0.7 – 1.1) | 0.9 (0.8 – 1.2) | 0.058 |
| HbA1c, % | 7.1 (6.3 – 7.9) | 6.7 (6.10 – 7.35) | **0.008** |
| Serum fasting glucose, mg/dL | 127 (108 – 150) | 131 (107 – 165.25) | 0.074 |
| TyG index | 4.65 (4.52 – 4.9) | 4.73 (4.59 – 4.95) | 0.651 |
| Total cholesterol, mg/dL | 164 (146.25 – 188) | 156 (131 – 190) | 0.091 |
| HDL-c, mg/dL | 48.5 (35 – 58.25) | 48 (41.75 – 57) | 0.937 |
| LDL-c, mg/dL | 85.4 (70.30 – 118.25) | 90 (68.15 – 113.25) | 0.689 |
| Triglycerides, mg/dL | 96 (72.5 – 134.5) | 92 (76 – 124) | 0.217 |
| AST, U/L | 50 (32 – 83.5) | 22 (16.75 – 28.25) | **< 0.001** |
| ALT, U/L | 60 (35.75 – 87.25) | 18.5 (13.75 – 27) | **< 0.001** |
| GGT, U/L | 44.5 (29 – 103.5) | 21 (15 – 40) | **< 0.001** |
| Total bilirubin, mg/dL | 0.73 (0.60 – 1.00) | 0.6 (0.5 – 1.00) | 0.365 |
| Microvascular complications, n | 21 (26.9) | 23 (29.5) | 0.72 |
| Macrovascular complications, n | 35 (44.9) | 39 (50) | 0.52 |
| Insulin therapy, n | 28 (35.9) | 23 (29.5) | 0.39 |
| Oral Hypoglycaemic therapies, n | 35 (44.9) | 31 (39.7) | 0.51 |
| Both, n | 6 (7.7) | 9 (11.5) | 0.41 |
| Lipid lowering therapies, n | 19 (24.4) | 38 (48.7) | **0.001** |

*Abbreviations: BMI (Body Mass Index), HbA1c (Glycated haemoglobin), TyG index (Triglyceride-Glucose index), HDL-c (high-density lipoprotein cholesterol), LDL-c (low-density lipoprotein cholesterol), AST (aspartate aminotransferase), ALT (alanine aminotransferase), GGT (gamma glutamyl transpeptidase).*

**Supplementary Table S4.** Univariate and multivariate regression analysis for the composite outcome of liver decompensation and hepatocellular carcinoma onset (liver disease progression).

| **Liver disease progression** | **Univariate model** | | | **Multivariate model** | | |
| --- | --- | --- | --- | --- | --- | --- |
| **Variables** | **OR** | **95% C.I.** | **p value** | **OR** | **95% C.I.** | **p value** |
| Age, years | 1.050 | 0.981 – 1.123 | 0.162 | **-** | **-** | **-** |
| Male gender | 0.782 | 0.237 – 2.580 | 0.686 | **-** | **-** | **-** |
| Liver cirrhosis | 777821127.4 | 0.000 – | 0.997 | **-** | **-** | **-** |
| BMI, kg/m^2^ | 1.034 | 0.926 – 1.155 | 0.553 | **-** | **-** | **-** |
| Liver stiffness, kPa | 1.058 | 1.013 – 1.105 | **0.011** | 1.061 | 0.976 – 1.154 | 0.166 |
| Albumin, g/dL | 0.087 | 0.015 – 0.508 | **0.007** | 0.010 | 0.000 – 2.209 | 0.094 |
| Gamma-globulins, g/dL | 2.472 | 0.886 – 6.903 | 0.084 | **-** | **-** | **-** |
| Creatinine, mg/dL | 0.920 | 0.113 – 7.493 | 0.938 | **-** | **-** | **-** |
| HbA1c, % | 1.226 | 0.786 – 1.912 | 0.369 | **-** | **-** | **-** |
| Serum fasting glucose, mg/dL | 1.000 | 0.987 – 1.014 | 0.957 | **-** | **-** | **-** |
| TyG index | 1.200 | 0.496 – 2.906 | 0.686 | **-** | **-** | **-** |
| Total cholesterol, mg/dL | 0.981 | 0.960 – 1.003 | 0.086 | **-** | **-** | **-** |
| Triglycerides, mg/dL | 1.000 | 0.992 – 1.009 | 0.992 | **-** | **-** | **-** |
| LDL-c, mg/dL | 0.971 | 0.944 – 0.998 | **0.037** | 0.938 | 0.883 – 0.998 | **0.041** |
| ALT, U/L | 0.996 | 0.984 – 1.009 | 0.583 | **-** | **-** | **-** |
| AST, U/L | 1.009 | 0.998 – 1.021 | 0.119 | **-** | **-** | **-** |
| GGT, U/L | 0.994 | 0.983 – 1.005 | 0.289 | **-** | **-** | **-** |
| Total bilirubin, mg/dL | 8.667 | 2.332 – 32.215 | **0.001** | 24.591 | 1.305 – 463.482 | **0.033** |
| Portal Vein diameter, mm | 1.838 | 1.195 – 2.826 | **0.006** | 1.460 | 0.615 – 3.464 | 0.391 |
| Spleen diameter, cm | 0.997 | 0.953 – 1.043 | 0.904 | **-** | **-** | **-** |
| Lipid lowering therapy | 0.919 | 0.225 – 3.757 | 0.906 | **-** | **-** | **-** |
| T2D therapy | 1.200 | 0.115 – 12.539 | 0.879 | **-** | **-** | **-** |
| PNPLA3 rs738409 I148 | 0.778 | 0.235 – 2.577 | 0.681 | **-** | **-** | **-** |
| TM6SF2 rs58542926 E167K | 0.559 | 0.100 – 3.139 | 0.509 | **-** | **-** | **-** |
| MBOAT7 rs641738 | 3.429 | 0.984 – 11.947 | **0.05** | 35.737 | 1.533 – 822.056 | **0.025** |

*Abbreviations: BMI (Body Mass Index), HbA1c (Glycated haemoglobin), TyG index (Triglyceride-Glucose index), HDL-c (high-density lipoprotein cholesterol), LDL-c (low-density lipoprotein cholesterol), AST (aspartate aminotransferase), ALT (alanine aminotransferase), GGT (gamma glutamyl transpeptidase), PNPLA3 (Patatin-like phospholipase domain-containing protein 3), TM6SF2 (Transmembrane 6 Superfamily Member 2), MBOAT7 (Membrane-bound O-acyltransferase 7).*

**Supplementary Table S5.** Univariate and multivariate regression analysis for the composite outcome of T2D vascular complications (micro- and macro-vascular complications).

| **T2D vascular complications** | **Univariate model** | | | **Multivariate model** | | |
| --- | --- | --- | --- | --- | --- | --- |
| **Variables** | **OR** | **95% C.I.** | **p value** | **OR** | **95% C.I.** | **p value** |
| Age, years | 0.997 | 0.948 – 1.049 | 0.918 | **-** | **-** | **-** |
| Male gender | 1.212 | 0.476 – 3.083 | 0.687 | **-** | **-** | **-** |
| Liver cirrhosis | 1.212 | 0.476 – 3.083 | 0.687 | **-** | **-** | **-** |
| BMI, kg/m^2^ | 0.942 | 0.859 – 1.034 | 0.212 | **-** | **-** | **-** |
| Liver stiffness, kPa | 1.016 | 0.978 – 1.056 | 0.407 | **-** | **-** | **-** |
| Albumin, g/dL | 0.488 | 0.144 – 1.655 | 0.250 | **-** | **-** | **-** |
| Gamma-globulins, g/dL | 1.579 | 0.615 – 4.055 | 0.324 | **-** | **-** | **-** |
| Creatinine, mg/dL | 0.994 | 0.195 – 5.060 | 0.994 | **-** | **-** | **-** |
| HbA1c, % | 1.030 | 0.698 – 1.520 | 0.881 | **-** | **-** | **-** |
| Serum fasting glucose, mg/dL | 1.004 | 0.992 – 1.015 | 0.529 | **-** | **-** | **-** |
| TyG index | 2.296 | 0.776 – 6.794 | 0.133 | **-** | **-** | **-** |
| Total cholesterol, mg/dL | 0.991 | 0.976 – 1.006 | 0.226 | **-** | **-** | **-** |
| Triglycerides, mg/dL | 1.004 | 0.995 – 1.012 | 0.397 | **-** | **-** | **-** |
| LDL-c, mg/dL | 0.992 | 0.976 – 1.008 | 0.339 | **-** | **-** | **-** |
| ALT, U/L | 1.002 | 0.993 – 1.011 | 0.658 | **-** | **-** | **-** |
| AST, U/L | 1.007 | 0.994 – 1.020 | 0.281 | **-** | **-** | **-** |
| GGT, U/L | 1.009 | 1.000 – 1.018 | 0.060 | **-** | **-** | **-** |
| Total bilirubin, mg/dL | 1.242 | 0.458 – 3.368 | 0.671 | **-** | **-** | **-** |
| Portal Vein diameter, mm | 1.093 | 0.801 – 1.490 | 0.575 | **-** | **-** | **-** |
| Spleen diameter, cm | 0.957 | 0.891 – 1.029 | 0.237 | **-** | **-** | **-** |
| Lipid lowering therapy | 0.879 | 0.300 – 2.580 | 0.815 | **-** | **-** | **-** |
| T2D therapy | 1.600 | 0.392 – 6.533 | 0.513 | **-** | **-** | **-** |
| PNPLA3 rs738409 I148 | 1.240 | 0.482 – 3.189 | 0.655 | **-** | **-** | **-** |
| TM6SF2 rs58542926 E167K | 2.043 | 0.468 – 8.914 | 0.342 | **-** | **-** | **-** |
| MBOAT7 rs641738 | 1.665 | 0.527 – 5.255 | 0.385 | **-** | **-** | **-** |

*Abbreviations: BMI (Body Mass Index), HbA1c (Glycated haemoglobin), TyG index (Triglyceride-Glucose index), HDL-c (high-density lipoprotein cholesterol), LDL-c (low-density lipoprotein cholesterol), AST (aspartate aminotransferase), ALT (alanine aminotransferase), GGT (gamma glutamyl transpeptidase), PNPLA3 (Patatin-like phospholipase domain-containing protein 3), TM6SF2 (Transmembrane 6 Superfamily Member 2), MBOAT7 (Membrane-bound O-acyltransferase 7)*.

**Supplementary Table S6.** Univariate and multivariate regression analysis for mortality.

| **Death** | **Univariate model** | | | **Multivariate model** | | |
| --- | --- | --- | --- | --- | --- | --- |
| **Variables** | **OR** | **95% C.I.** | **p value** | **OR** | **95% C.I.** | **p value** |
| Age, years | 1.052 | 0.968 – 1.145 | 0.234 | **-** | **-** | **-** |
| Male gender | 2.625 | 0.642 – 10.728 | 0.179 | **-** | **-** | **-** |
| Liver cirrhosis | 1.867 | 0.480 – 7.255 | 0.368 | **-** | **-** | **-** |
| BMI, kg/m^2^ | 1.057 | 0.946 – 1.182 | 0.330 | **-** | **-** | **-** |
| Liver stiffness, kPa | 1.018 | 0.970 – 1.070 | 0.468 | **-** | **-** | **-** |
| Albumin, g/dL | 0.176 | 0.022 – 1.390 | 0.099 | **-** | **-** | **-** |
| Gamma-globulins, g/dL | 1.269 | 0.442 – 3.648 | 0.658 | **-** | **-** | **-** |
| Creatinine, mg/dL | 3.545 | 0.349 – 35.990 | 0.284 | **-** | **-** | **-** |
| HbA1c, % | 1.100 | 0.691 – 1.751 | 0.687 | **-** | **-** | **-** |
| Serum fasting glucose, mg/dL | 0.988 | 0.968 – 1.009 | 0.259 | **-** | **-** | **-** |
| TyG index | 0.615 | 0.226 – 1.671 | 0.341 | **-** | **-** | **-** |
| Total cholesterol, mg/dL | 1.008 | 0.985 – 1.032 | 0.510 | **-** | **-** | **-** |
| Triglycerides, mg/dL | 1.003 | 0.993 – 1.012 | 0.561 | **-** | **-** | **-** |
| LDL-c, mg/dL | 1.015 | 0.987 – 1.044 | 0.298 | **-** | **-** | **-** |
| ALT, U/L | 0.997 | 0.986 – 1.008 | 0.572 | **-** | **-** | **-** |
| AST, U/L | 0.987 | 0.965 – 1.009 | 0.258 | **-** | **-** | **-** |
| GGT, U/L | 0.997 | 0.987 – 1.007 | 0.554 | **-** | **-** | **-** |
| Total bilirubin, mg/dL | 1.528 | 0.319 – 7.309 | 0.595 | **-** | **-** | **-** |
| Portal Vein diameter, mm | 0.940 | 0.612 – 1.444 | 0.778 | **-** | **-** | **-** |
| Spleen diameter, cm | 1.080 | 0.881 – 1.325 | 0.457 | **-** | **-** | **-** |
| Lipid lowering therapy | 0.675 | 0.145 – 3.135 | 0.616 | **-** | **-** | **-** |
| T2D therapy | 0.722 | 0.062 – 8.484 | 0.723 | **-** | **-** | **-** |
| PNPLA3 rs738409 I148 | 1.000 | 0.254 – 3.929 | 1.000 | **-** | **-** | **-** |
| TM6SF2 rs58542926 E167K | 0.458 | 0.057 – 3.69. | 0.464 | **-** | **-** | **-** |
| MBOAT7 rs641738 | 1.206 | 0.280 – 5.206 | 0.801 | **-** | **-** | **-** |

*Abbreviations: BMI (Body Mass Index), HbA1c (Glycated haemoglobin), TyG index (Triglyceride-Glucose index), HDL-c (high-density lipoprotein cholesterol), LDL-c (low-density lipoprotein cholesterol), AST (aspartate aminotransferase), ALT (alanine aminotransferase), GGT (gamma glutamyl transpeptidase), PNPLA3 (Patatin-like phospholipase domain-containing protein 3), TM6SF2 (Transmembrane 6 Superfamily Member 2), MBOAT7 (Membrane-bound O-acyltransferase 7)*.

**Supplementary Table S7.** Univariate and multivariate regression analysis for the composite outcome of general progression (T2D vascular complications and liver disease progression).

| **General progression** | **Univariate model** | | | **Multivariate model** | | |
| --- | --- | --- | --- | --- | --- | --- |
| **Variables** | **OR** | **95% C.I.** | **p value** | **OR** | **95% C.I.** | **p value** |
| Age, years | 1.016 | 0.954 – 1.071 | 0.551 | **-** | **-** | **-** |
| Male gender | 1.077 | 0.412 – 2.818 | 0.880 | **-** | **-** | **-** |
| Liver cirrhosis | 1.750 | 0.661 – 4.630 | 0.260 | **-** | **-** | **-** |
| BMI, kg/m^2^ | 0.946 | 0.851 – 1.040 | 0.252 | **-** | **-** | **-** |
| Liver stiffness, kPa | 1.047 | 0.995 – 1.101 | 0.074 | **-** | **-** | **-** |
| Albumin, g/dL | 0.362 | 0.098 – 1.331 | 0.126 | **-** | **-** | **-** |
| Gamma-globulins, g/dL | 1.712 | 0.631 – 4.646 | 0.291 | **-** | **-** | **-** |
| Creatinine, mg/dL | 1.411 | 0.252 – 7.886 | 0.695 | **-** | **-** | **-** |
| HbA1c, % | 0.915 | 0.621 – 1.349 | 0.654 | **-** | **-** | **-** |
| Serum fasting glucose, mg/dL | 1.003 | 0.991 – 1.014 | 0.645 | **-** | **-** | **-** |
| TyG index | 1.247 | 0.694 – 2.240 | 0.460 | **-** | **-** | **-** |
| Total cholesterol, mg/dL | 0.987 | 0.972 – 1.003 | 0.112 | **-** | **-** | **-** |
| Triglycerides, mg/dL | 1.003 | 0.994 – 1.011 | 0.534 | **-** | **-** | **-** |
| LDL-c, mg/dL | 0.990 | 0.973 – 1.007 | 0.242 | **-** | **-** | **-** |
| ALT, U/L | 1.004 | 0.994 – 1.014 | 0.437 | **-** | **-** | **-** |
| AST, U/L | 1.013 | 0.998 – 1.029 | 0.094 | **-** | **-** | **-** |
| GGT, U/L | 1.010 | 0.999 – 1.020 | 0.067 | **-** | **-** | **-** |
| Total bilirubin, mg/dL | 1.310 | 0.460 – 3.733 | 0.613 | **-** | **-** | **-** |
| Portal Vein diameter, mm | 1.143 | 0.826 – 1.583 | 0.420 | **-** | **-** | **-** |
| Spleen diameter, cm | 0.958 | 0.897 – 1.024 | 0.204 | **-** | **-** | **-** |
| Lipid lowering therapy | 0.694 | 0.233 – 2.062 | 0.511 | **-** | **-** | **-** |
| T2D therapy | 2.609 | 0.273 – 24.940 | 0.405 | **-** | **-** | **-** |
| PNPLA3 rs738409 I148 | 1.330 | 0.503 – 3.513 | 0.565 | **-** | **-** | **-** |
| TM6SF2 rs58542926 E167K | 2.500 | 0.569 – 10.980 | 0.225 | **-** | **-** | **-** |
| MBOAT7 rs641738 | 1.330 | 0.418 – 4.234 | 0.629 | **-** | **-** | **-** |

*Abbreviations: BMI (Body Mass Index), HbA1c (Glycated haemoglobin), TyG index (Triglyceride-Glucose index), HDL-c (high-density lipoprotein cholesterol), LDL-c (low-density lipoprotein cholesterol), AST (aspartate aminotransferase), ALT (alanine aminotransferase), GGT (gamma glutamyl transpeptidase), PNPLA3 (Patatin-like phospholipase domain-containing protein 3), TM6SF2 (Transmembrane 6 Superfamily Member 2), MBOAT7 (Membrane-bound O-acyltransferase 7).*
